# Understanding cold stress response mechanisms in plants: an overview

**DOI:** 10.3389/fpls.2024.1443317

**Published:** 2024-11-06

**Authors:** Zhenfeng Qian, Lilian He, Fusheng Li

**Affiliations:** ^1^ College of Agronomy and Biotechnology, Yunnan Agricultural University, Kunming, China; ^2^ The Key Laboratory for Crop Production and Smart Agriculture of Yunnan Province, Yunnan Agricultural University, Kunming, China

**Keywords:** cold stress, cold signal perception and transduction, ICE1-CBF-COR transcription cascade, ROS homeostasis, plant hormone signal

## Abstract

Low-temperature stress significantly impacts plant growth, development, yield, and geographical distribution. However, during the long-term process of evolution, plants have evolved complicated mechanisms to resist low-temperature stress. The cold tolerance trait is regulated by multiple pathways, such as the Ca^2+^ signaling cascade, mitogen-activated protein kinase (MAPK) cascade, inducer of CBF expression 1 (ICE1)-C-repeat binding factor (CBF)-cold-reulated gene (COR) transcriptional cascade, reactive oxygen species (ROS) homeostasis regulation, and plant hormone signaling. However, the specific responses of these pathways to cold stress and their interactions are not fully understood. This review summarizes the response mechanisms of plants to cold stress from four aspects, including cold signal perception and transduction, ICE1-CBF-COR transcription cascade regulation, ROS homeostasis regulation and plant hormone signal regulation. It also elucidates the mechanism of cold stress perception and Ca^2+^ signal transduction in plants, and proposes the important roles of transcription factors (TFs), post-translational modifications (PTMs), light signals, circadian clock factors, and interaction proteins in the ICE1-CBF-COR transcription cascade. Additionally, we analyze the importance of ROS homeostasis and plant hormone signaling pathways in plant cold stress response, and explore the cross interconnections among the ICE1-CBF-COR cascade, ROS homeostasis, and plant hormone signaling. This comprehensive review enhances our understanding of the mechanism of plant cold tolerance and provides a molecular basis for genetic strategies to improve plant cold tolerance.

## Introduction

Low-temperature stress significantly affects the growth, development, yield and geographical distribution of plants (Adhikari et al., 2022; Guan et al., 2023). Accumulating evidence indicates that low temperature can increase membrane permeability, impair photosynthesis, cause oxidative damage, and even lead to abundant cell death (Orvar et al., 2000; Lukatkin, 2003; Theocharis et al., 2012; Uritani et al., 2014; Liu et al., 2018). In addition, continuous low-temperature stress can cause severe damage to plants and even lead to death. In the process of crop production, low-temperature stress inhibits seed vigor and the germination rate, slows crop growth, and decreases crop yield and quality (Rutayisire et al., 2020; Xu et al., 2022). However, during the long-term process of evolution, plants have evolved complicated mechanisms to tolerate low-temperature stress.

The freezing tolerance of many plants can be increased by cold acclimation, a process involving preexposure to low, nonfreezing temperatures (Zhao et al., 2016; Qi et al., 2022). Cold acclimation is a multifaceted process that encompasses the perception and transduction of environmental cold stress signals, the expression of cold tolerance genes, and specific metabolic and physiological changes (Xiong et al., 2002; Zhu, 2016; Chang et al., 2020). Usually, cold receptors on the plant cell membrane sense low-temperature signals and trigger calcium influx to activate the calcium (Ca^2+^) signaling cascade network (Ma et al., 2015) and the phosphorylation cascade regulated by protein kinases (Zhao et al., 2017), ultimately activating the response of the downstream ICE1-CBF-COR signaling cascade pathway (Gusain et al., 2023). The ICE1-CBF-COR pathway is considered the most important and archetypal cold stress response pathway in plants. However, the transcriptional activation of the ICE1-CBF-COR cascade is a complex network involving direct or indirect regulation of transcription factors (TFs) (Zhang et al., 2022c; Kamble, 2024), post-translational modifications (PTMs) (Zhao et al., 2017; Ding et al., 2019), light signals (Jiang et al., 2020; Zhang et al., 2020a), circadian clock factors (Kidokoro et al., 2021, Kidokoro et al., 2023) and interacting proteins (Jin et al., 2017; Lee et al., 2021).

In general, plant cells improve cold tolerance by accumulating osmotic regulators such as proline (Pro), soluble sugar (Ss), and soluble protein (Sp), as well as increasing the activities of antioxidant enzymes including catalase (CAT), superoxide dismutase (SOD), ascorbate peroxidase (APX), and peroxidase (POD) (Yan et al., 2019; Ritonga and Chen, 2020; Zhang et al., 2020b; Ramazan et al., 2021). These changes in physiological activities help maintain cell behavior and activity, protect against damage from reactive oxygen species (ROS), and maintain the stability of biologically active cell membranes and protein structures (Ritonga and Chen, 2020). TFs such as basic leucine zipper (bZIP) (Bai et al., 2022), myeloblastosis (MYB) (Yang et al., 2022a) and teosinte branched 1/cycloidea/proliferating cell factor (TCP) (Li et al., 2022b) bind to the promoters of genes encoding antioxidant enzymes, thereby increasing their expression and ultimately enhancing plant cold tolerance. Interestingly, maintaining appropriate ROS levels is essential for plant cold tolerance. Because ROS also act as signaling molecule that activate *CBF* transcription (Liu et al., 2022), this suggests an interaction between ROS homeostasis and the CBF signaling pathway.

Plant hormones, including abscisic acid (ABA) (Hauser et al., 2017), salicylic acid (SA) (Wang et al., 2020), strigolactones (SL) (Cooper et al., 2018), brassinosteroid (BR) (An et al., 2023), ethylene (ETH) (Wang et al., 2021a) and jasmonic acid (JA) (Hu et al., 2017), also play important roles in plant growth and development and stress response. Cold stress can promote the biosynthetic pathway of these hormones, thereby activating their signaling pathways to respond to cold stress. In addition, there are also interactions between these hormone signaling pathways, such as brassinazole resistant 1 (BZR1, a positive regulator of the BR signaling pathway) enhances 9-cis epoxycarotenoid dioxygenase (*NCED1*, a key enzyme for the process of ABA synthesis) gene expression and increases the level of ABA (An et al., 2023), thereby increasing cold tolerance in tomato plants. Interestingly, in these plant hormone signaling pathways, ABA regulation of cold tolerance is CBF-independent (Li et al., 2021), whereas BR, SA, SL, ETH, and JA depend on CBF (Ye et al., 2019; Wang et al., 2020, Wang et al., 2021a, Wang et al., 2023b; Yang et al., 2023). In addition, ABA, SA and SL also promote ROS scavenging and regulate cold tolerance (Pan et al., 2020; Li et al., 2021; Wang et al., 2023b). These studies revealed interactions among the plant hormone signaling pathways, *CBF* transcription and ROS homeostasis.

**Figure 1:**
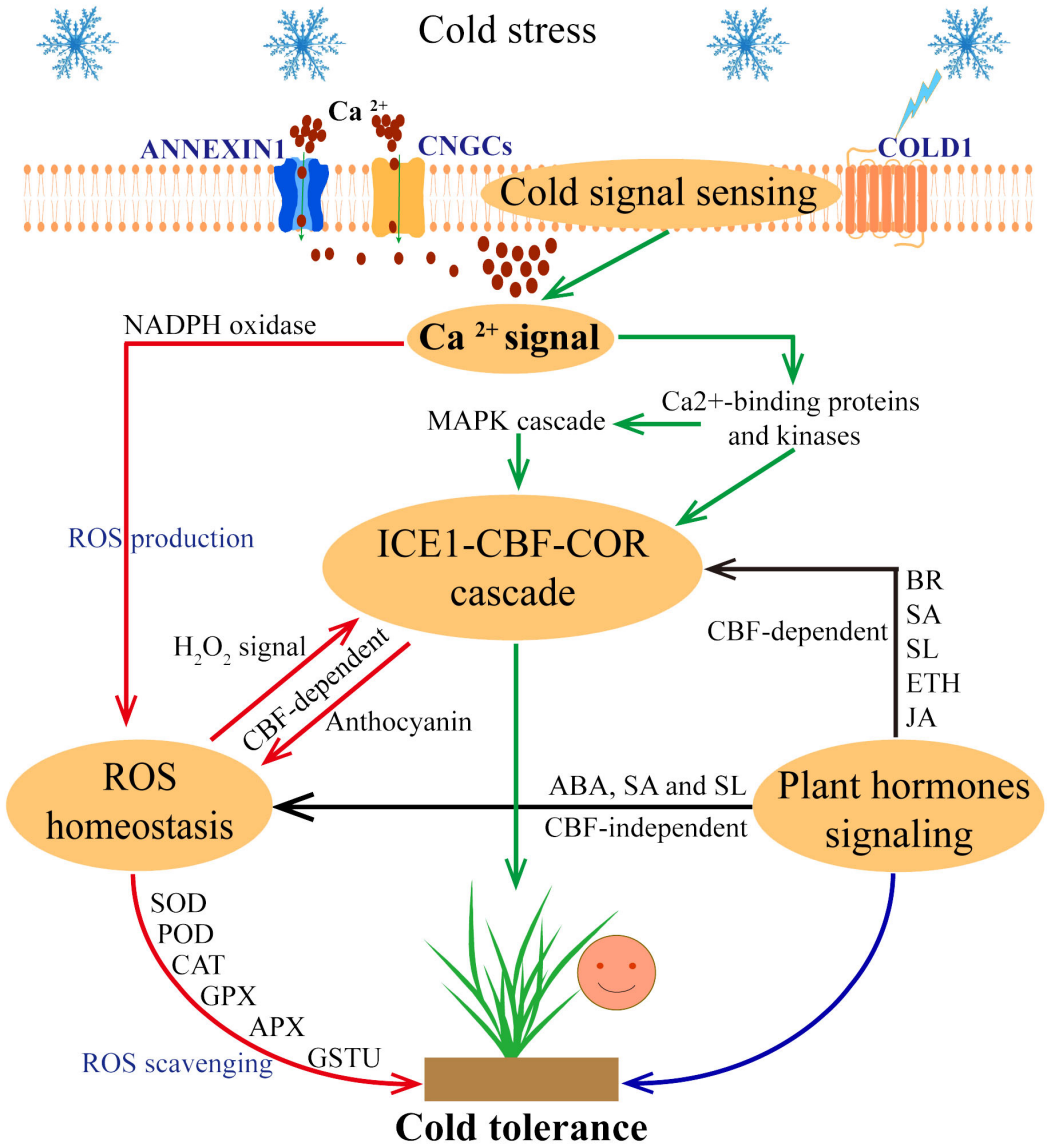
Response strategies of plants to cold stress. When cold stress occurs, plant cells can sense and transduce cold signals, COLD1 senses low temperature and triggers Ca^2+^ channels (ANNEXIN1 and CNGCs) to transport Ca^2+^ into the cytoplasm. Further, Ca^2+^ signaling pathway activates the ICE1-CBF-COR cascade (through the Ca^2+^-binding proteins and kinases, MAPK cascade pathway) and ROS homeostasis (through the NADPH oxidase pathway). In this process, ICE1-CBF-COR cascade and ROS homeostasis are involved in CBF-dependent ways to respond to cold stress. In addition, plant hormone signaling pathways also regulate plant cold tolerance through CBF-dependence and ROS homeostasis, indicating a cross interconnections between these pathways.

Although the functions and regulatory mechanisms of many cold tolerance-related genes have been revealed, there is still a lack of systematic reviews on how to integrate these new ideas into the complex mechanism network of the plant response to low temperature. In this review, we summarize four aspects of the response mechanism of plants to low-temperature stress (I. Plant perception and transduction of cold stress signals; II. ICE1-CBF-COR transcription cascade regulation; III. ROS homeostasis regulation; and IV. plant hormone signal regulation), and presents the cross interconnections between these pathways (**Figure 1**). This comprehensive overview enhances our understanding of plant cold tolerance mechanisms and offers insights for genetic improvements in enhancing plant cold tolerance.

## Cold signal perception and transduction

**Figure 2:**
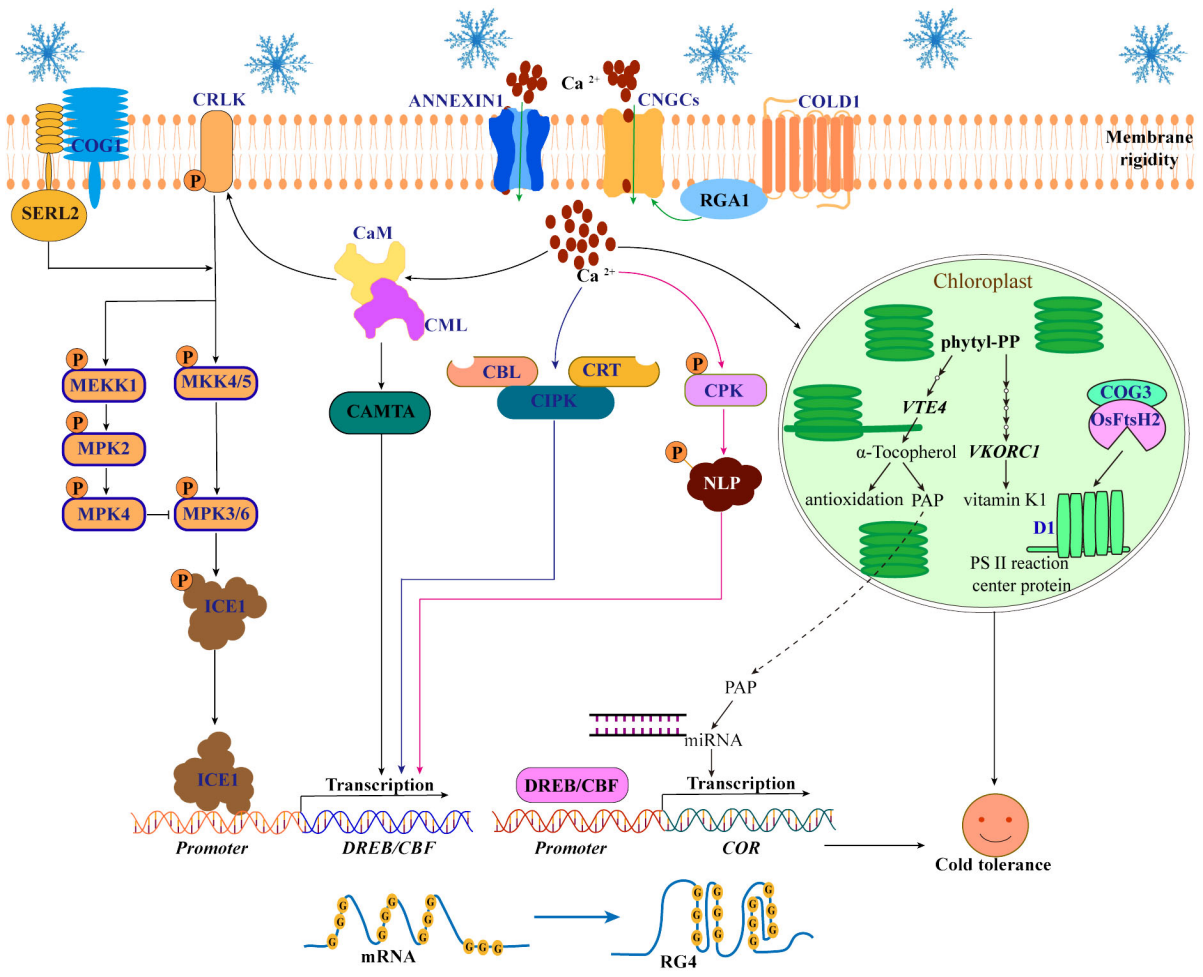
Perception and transduction of cold signals in plants. When cold stress occurs, the COLD1 senses cold signals and then interacts with RGA1 to activate Ca^2+^ channels (ANNEXIN1 and CNGCs) to release Ca^2+^ into the cytoplasm. An increase in Ca^2+^ in the cytoplasm can further activate the regulatory pathways of three Ca^2+^-binding proteins (CaM/CML, CBLs and CDPK/CPK) to enhance the cold tolerance of plants. First, CaM/CML activates CRLK1 and CAMTA to regulate the MAPK-ICE1-CBF cascade pathway. Second, CBLs and CRT interact with CIPKs to increase the expression levels of *DREB/CBF*, respectively. Third, CPK phosphorylates NLP7 and promotes its ability to regulate the expression of CBFs. In addition, Ca^2+^ signaling triggered by COLD1 promotes the biosynthesis pathway of vitamins E and K (By increasing the expression of *VTE4* and *VKORC1*), leading to the production of PAP and its transport into the nucleus to regulate the miRNAs in chloroplasts. Moreover, cold-induced COG3 interaction with OsFtsH2 maintains the balance of photosystem II protein D1 in chloroplasts. Interestingly, the RG4 structure of mRNA in the nucleus and the COG1-OsSERL2 complex in the plasma membrane also function as cold receptors.

Plants sense low-temperature stress signals, which in turn regulate downstream Ca^2+^ signaling networks and activate the expression of cold-responsive genes (**Figure 2**). When plants are exposed to low temperature, chilling-tolerance divergence 1 (COLD1) perceives cold signals, subsequently interacting with G-protein α subunit 1 (RGA1) to activate Ca^2+^ channels and trigger Ca^2+^ influx (Ma et al., 2015; Shi and Yang, 2015). Current studies have reported that some Ca^2+^ channel proteins are activated by cold stress, including cyclic nucleotide-gated channels (CNGCs), Ca^2+^-permeable transporter (ANNEXIN1), mid1 complementing activity (MCA1) and mid2 complementing activity (MCA2), which mediate Ca^2+^ transport and positively regulate plant cold tolerance (Wang et al., 2021b; Peng et al., 2024; Mori et al., 2018; Liu et al., 2021). It is well known that at normal temperatures, the activity of these Ca^2+^ channel proteins is inhibited to maintain lower concentrations of Ca^2+^ in plant cells, and only when cold stress occurs are they activated to increase the Ca^2+^ influx. Therefore, it is necessary to further understand the mechanism by which cold stress activates Ca^2+^ channel protein activity. Interestingly, recent studies have suggested that the activity of CNGCs is activated by the phosphorylation of plant peptide containing sulfated tyrosine1 receptor (PSY1R) and osmotic stress/aba-activated protein kinase 8 (OsSAPK8) under cold stress (Wang et al., 2021b; Peng et al., 2024). In addition, Ca^2+^ transport activity of ANNEXIN1 was activated by OST phosphorylation under cold stress (Liu et al., 2021). However, CNGC20 is degraded by cold-responsive protein kinase 1 (CRPK1)-mediated phosphorylation under long-term cold stress. Researchers believe that Ca^2+^ transport mediated by Ca^2+^ channel proteins is an early event in the plant response to cold stress (Peng et al., 2024). Therefore, it is worth considering how plants in long-term snow-covered areas maintain the transmission of cold signals, which clearly requires further research to reveal.

As a secondary messenger, Ca^2+^ plays an important role in the transduction of cold signals in plants (Liu et al., 2021). The increase of Ca^2+^ levels in cytoplasmic can further activate the regulatory pathways of three Ca^2+^-binding proteins (calmodulin/calmodulin-like protein (CaM/CML) (Yang et al., 2010a), Ca^2+^ binding to calcineurin B-like protein (CBL) (Huang et al., 2011) and calcium-dependent protein kinase (CDPK/CPK) (Ding et al., 2022)) to increase the cold tolerance (**Figure 2**). First, Ca^2+^ binds to CaM and CML to activate Ca^2+^/CaM-regulated receptor-like kinase 1 (CRLK1) (Yuan et al., 2018) and calmodulin-binding transcription activator (CAMTA) (Doherty et al., 2009). In *Arabidopsis*, CRLK1 regulates the mitogen-activated protein kinases (MAPK) cascade pathway: CRLK1 phosphorylation activates the MKK4/5-MPK3/6 cascade to promote the degradation of ICE1 and negatively regulates cold tolerance. However, CRLK1 phosphorylation activates the MEKK1-MKK2-MPK4 cascade to inhibit MPK3/6 phosphorylation to degrade ICE1 and positively regulate cold tolerance (Yang et al., 2010b; Zhao et al., 2017; Duan et al., 2018. In addition, CaM/CML promotes CAMTA3 binding to the DNA motifs (CM2) cis-element on the *CBF* promoter to positively regulate the expression of *CBF* and increase cold tolerance (Doherty et al., 2009; Kim et al., 2013). Second, CBL promotes interactions with CBL protein kinases (CIPKs) to increase the expression levels of *DREB/CBF* and enhance cold tolerance in *Arabidopsis* (Huang et al., 2011). Moreover, calreticulin 3 (OsCRT3) conformational changes to promote interaction with OsCIPK7 play an important role in increasing the expression levels of *CBF* in rice (Zhang et al., 2019a; Guo et al., 2023). Third, Ca^2+^ binds to CDPK/CPK to increase the CPK phosphorylation of nin-like protein 7 (NLP7) and promotes the transfer of NLP7 from the cytoplasm to the nucleus to regulate the expression of the *CBF* and *COR* genes, increasing cold tolerance in *Arabidopsis* (Ding et al., 2022).

In addition to activating pathways regulated by Ca^2+^-binding proteins, Ca^2+^ also regulates the cold response pathway in chloroplasts. For example, Ca^2+^ signaling triggered by COLD1 increases the expression of *VTE4* (the gene responsible for α-tocopherol (vitamin E)) and *VKORC1* (the key gene in the vitamin K1 pathway) and promotes the biosynthesis pathway of vitamins E and K in chloroplasts, leading to the production of 3′-phosphoadenosine 5′-phosphate (PAP) and its transport into the nucleus to regulate the production of miRNAs, ultimately promoting the expression of cold-responsive genes and improving cold tolerance in rice (Luo et al., 2021). However, the mechanism by which Ca^2+^ regulates the vitamin network remains unclear. Additionally, chilling-tolerance in Geng/japonica rice 3 (COG3), a putative calmodulin-binding protein, interacts with the proteolytic enzyme OsFtsH2 to remove the cold-damaged photosystem II protein D1 and balance the turnover of the D1 protein in chloroplasts, thereby maintaining photosynthetic efficiency and regulating cold tolerance in rice (Liu et al., 2024). However, it is not clear whether the function of the COG3 is regulated by Ca^2+^ signaling pathway. Therefore, further elucidation of the mechanism by which Ca^2+^ signaling activates the cold response pathway in chloroplasts is warranted.

COLD1 is a low-temperature sensor that activates the early Ca^2+^ signaling network. However, a single low-temperature sensor is not enough to realize the sensing and transduction of cold signals in complex networks. Other cold sensors have also been identified. In *Arabidopsis*, cold stress triggers the folding of guanine (G-rich) sequences of mRNAs into RNA G-quadruplex (RG4) structures, which inhibits mRNA degradation and increases cold tolerance (Yang et al., 2022b). The author proposed that the RG4 structure, as a low-temperature sensor, can maintain the stability of mRNAs under cold stress. This study demonstrated the importance of the RG4 structure in plant cold tolerance, which is helpful for the excavation and utilization of cold-tolerant germplasm resources. However, we hypothesized whether the RG4 structure is related to the physiological activity and structure of protoplasts under cold stress. In addition, the chilling tolerance in Gengdao 1 (COG1) protein, which is induced by cold stress, interacts with somatic embryogenesis receptor-like kinase 2 (OsSERL2) to form the COG1-OsSERL2 complex in the cell membrane. This complex subsequently activates the MAPK signaling cascade, contributing to enhanced cold tolerance in rice (Changxuan et al., 2023). Both the COG1-OsSERL2 complex and COLD1 are membrane-localized low-temperature sensors that together promote the MAPK cascade, but the COG1-OsSERL2 complex is independent of the Ca^2+^ signaling pathway (**Figure 2**).

## The ICE1-CBF-COR transcriptional cascade enhances plant cold tolerance

The ICE1-CBF-COR transcriptional cascade, a key component of the downstream COLD1-Ca^2+^-binding protein-MAPK signaling cascade, is widely recognized as the prototypical and evolutionarily conserved response pathway to cold stress in plants. In general, the transcriptional activity of the ICE1-CBF-COR cascade in cold-tolerant germplasms is greater than that in cold-sensitive germplasms (Todorovska et al., 2014). ICE1, a MYC-like basic helix-loop-helix (bHLH) transcription factor, serves as the central regulator of cold-responsive gene expression (Wang et al., 2023c). In *Arabidopsis*, ICE1/2 binds to the MYC binding site (CANNTG) in the *CBF1/2/3* gene promoter, leading to the upregulation of the *CBF1/2/3* gene, which in turn controls the transcription of *COR* genes and positively regulates cold tolerance (Kim et al., 2015). This transcriptional cascade also plays a role in enhancing cold tolerance in transgenic plants of rice (Verma et al., 2020), *Zea mays* (Lu et al., 2017) and *Zoysia japonica* (Zuo et al., 2019). Furthermore, ICE1 not only regulates *CBF* but can also directly interacts with the *COR* gene promoter to facilitate *COR* gene transcription (Tang et al., 2020).

The dehydration-responsive element binding protein/C-repeat binding factors DREB/CBFs belong to the APETALA2/ethylene- responsive element binding factor (AP2/ERF) superfamily of TFs and are pivotal in the ICE1-CBF-COR cascade pathway. In *Arabidopsis* and rice, *DREB1A/CBF3*, *DREB1B/CBF1*, and *DREB1C/CBF2* are known as typical cold-inducible genes that are crucial for plant cold stress resistance (Liu et al., 1998; Ito et al., 2006; Zhao et al., 2016). Approximately 10-20% of *COR* genes in *Arabidopsis* are estimated to be directly regulated by CBFs (Kosova et al., 2021), which include low temperature-induced (*LTI*), cold-inducible (*KIN*), response-to-dehydration (*RD*) and early dehydration inducible (*ERD*) gene families (Thomashow, 1998). These families include *COR78*, *COR15*, *COR6.6*, and *COR47*, with *COR47* encoding late embryogenesis-abundant (LEA) family homologous proteins dehydrin (DHN) (Thomashow, 1998). The expression of these *COR* genes results in the production of hydrophilic peptides that protect cells from freezing damage. In addition, many studies have indicated that the overexpression of these *COR* genes in transgenic plants increases cold tolerance by scavenging ROS and increasing membrane stability (Vladan et al., 2013; Liu et al., 2015; Guo et al., 2019; Chen et al., 2022). These studies demonstrated the importance of the ICE1-CBF-COR transcriptional cascade in the plant cold stress response. Therefore, identification of the transcriptional activity of the ICE1-CBF-COR transcriptional cascade can be used as molecular evidence for the evaluation of cold-tolerant germplasms.

## Regulation of the ICE1-CBF-COR transcriptional cascade

Recent studies revealed that the ICE1-CBF-COR cascade pathway is regulated by various TFs (**Table 1**). These TFs directly target the ICE1-CBF-COR transcriptional cascade and regulate cold tolerance in plants. For example, MYBs not only bind to the promoter of *ICE1* but also interact with the promoter of *CBFs* to increase the transcription level of *ICE1* and *CBFs* (An et al., 2018; Jiang et al., 2022). In addition, the B-box (BBX), NAC (no apical meristem (NAM), *Arabidopsis* transcription activation factor (ATAF) and cup-shaped cotyledon (CUC)), WRKY and basic leucine zipper (bZIP) TFs are also involved in regulating *CBF* transcription (Hao et al., 2011; An et al., 2021; Zhang et al., 2022c; Kamble, 2024). Moreover, some TFs related to light signaling (phytochrome interacting factors (PIF) and phytochrome B (phyB)), circadian clock (REVEILLE4 (RVE) and pseudoresponse regulators (PRRs)), and plant hormone signaling (brassinazole resistant 1 (BZR1), jasmonate zim-domain 1 (JAZ1) and ethylene-insensitive 3 (EIN3)) also play a role in regulating the ICE1-CBF-COR transcriptional cascade (**Table 1**). Therefore, the transcription of the ICE1-CBF-COR cascade involves a complex network involving many TFs and regulatory pathways. Further analysis of the promoter functional elements will help to elucidate the transcriptional regulatory network upstream of the ICE1-CBF-COR cascade.

**Table 1:** Transcription factors targeting the ICE1-CBF-COR transcriptional cascade.

TFs	Regulation function	Reference
AtMYB43	Interacts with ICE1 to negatively regulate *CBF*	(Zheng et al., 2023)
ZmMYB39	Positively regulates *ICE1*	(Jiang et al., 2022)
MdBBX37	Interacts with ICE1 to positively regulate *CBF*	(An et al., 2021)
SlBBX17	Interacts with HY5 to positively regulate *CBF*	(Song et al., 2023)
VaMYC2	Activates the expression of *VaCBF1*	(Hu et al., 2022)
MdMYB23	Positively regulates *CBF*	(An et al., 2018)
GmNAC20	Increases the transcription level of *DREB1A/CBF3*	(Hao et al., 2011)
MdNAC104	Promotes the expression of *CBF*	(Mei et al., 2023)
CdWRKY2	Activates the expression of *VaCBF1*	(Huang et al., 2022)
OsWRKY76	Increases the transcription level of *OsDREB1*	(Zhang et al., 2022c)
OsbZIP46	Increases the transcription level of *OsDREB1*	(Kamble, 2024)
ZmbZIP68	Negatively regulates the expression of *DREB1*	(Zhuoyang et al., 2022)
AtPIF3	Downregulates the expression of *CBF*	(Jiang et al., 2017)
AtphyB	Enhances the expression of *COR* gene	(Jiang et al., 2020)
AtRVE4/8	Activates the expression of *DREB1s*	(Kidokoro et al., 2021)
AtPRR	Negatively regulates the expression of *CBFs*	(Nakamichi et al., 2009)
PbeNAC1	Interacts with DREB1 to enhance the expression of *CORs*	(Jin et al., 2017)
AtBZR1	Positively regulates *CBF* expression	(Li et al., 2017)
AtJAZ1	Interacts with ICE1 to inhibit the transcriptional activity of *ICE1*	(Hu et al., 2013)
AtEIN3	Negatively regulates the expression of *CBFs*	(Shi et al., 2012)

**Figure 3:**
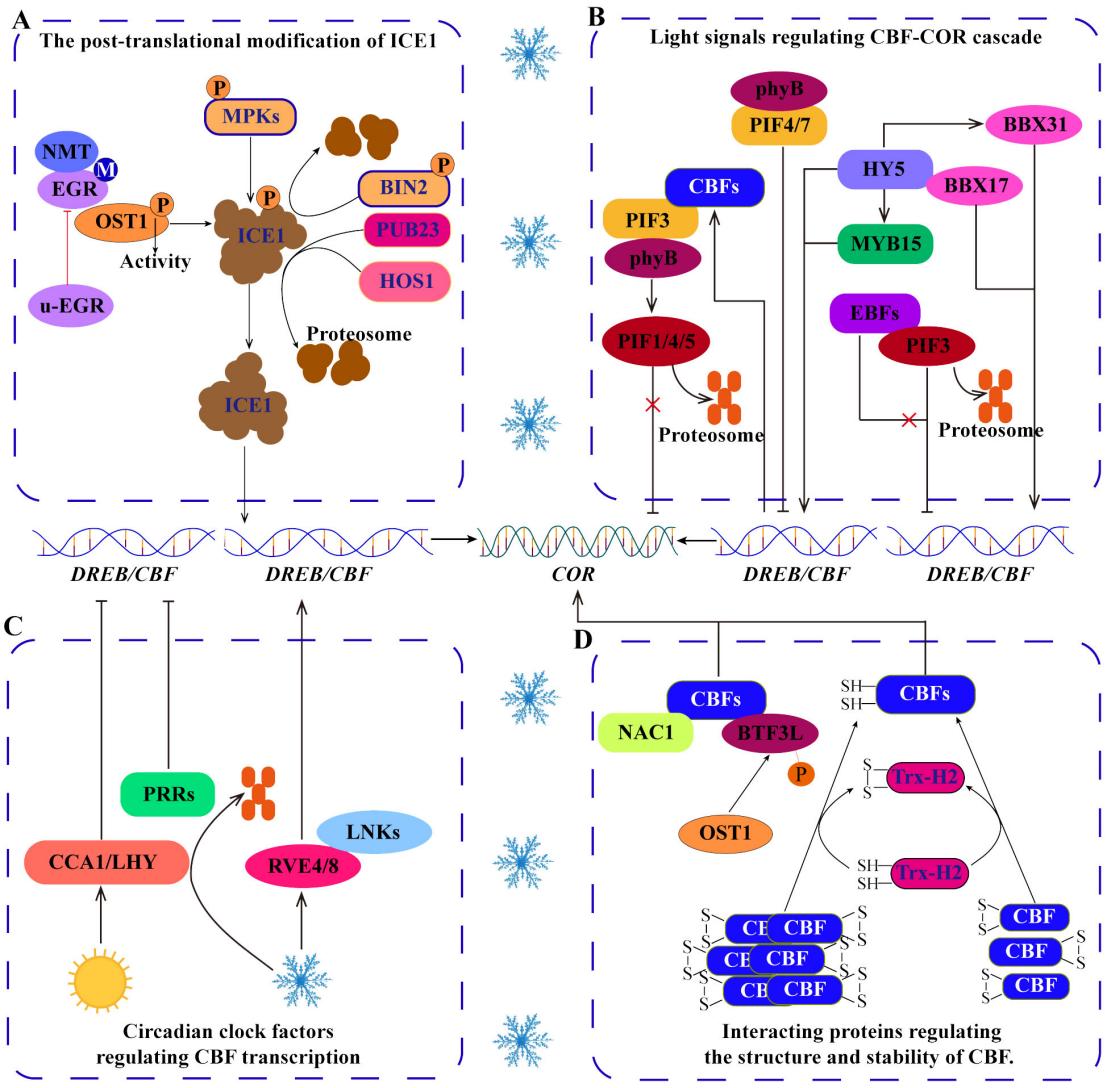
Regulation of the ICE1-CBF-COR signaling cascade. **(A)** Posttranslational modifications, including phosphorylation, myristoylation and ubiquitination, regulate the stability of the ICE1 protein under cold stress. **(B)** Light signal pathway TFs regulate the CBF-COR cascade. **(C)** Circadian clock factors regulate the expression of *CBFs*. **(D)** Some proteins interact with CBFs to regulate their structure and stability.

Post-translational modifications (PTMs), including phosphorylation, myristoylation and ubiquitination, can change the structure, stability, activity and function of proteins, which is an important reason for protein diversity. These modifications can generally be induced by cold stress and directly or indirectly regulate the ICE1-CBF-COR transcriptional cascade (Ding et al., 2019; Ye et al., 2019; Wang et al., 2022) (**Figure 3A**). For example, the MPK cascade phosphorylates ICE1 to regulate the stability of ICE1, which is very important for the response of *Arabidopsis* and rice to cold stress (Zhao et al., 2017). Independent of the MPK cascade, open stomata 1 (OST1) phosphorylates ICE1 to increase its stability, whereas brassinosteroid insensitive 2 (BIN2) phosphorylates ICE1 to promote its degradation (Zhan et al., 2015; Ye et al., 2019). Notably, the phosphorylation of ICE1 by OST1 is regulated by myristoylation and ubiquitination, which may be important strategies for balanced plant growth and cold tolerance. Under normal temperature, clade-E growth-regulating 2 (EGR2) is N‐myristoylated by N-myristoyltransferase 1 (NMT1) to form m-EGR2. The interaction between m-EGR2 and OST1 inhibits the activity of OST1 kinase, which is beneficial for the normal growth of plants. However, under cold stress, EGR2 myristoylation is suppressed, leading to the dominance of unmyristoylated EGR2 (u-EGR2). u-EGR2 inhibits interaction between m-EGR2 and OST1 increasing OST1 activity, facilitating ICE1 phosphorylation by OST1, and maintaining the stability of ICE1 to enhance cold tolerance in *Arabidopsis* (Ding et al., 2019). In addition, the E3 ubiquitin ligase HOS1 (high expression of osmotically responsive gene 1) can promote ICE1 degradation in *Arabidopsis*, but this process is inhibited by OST1 under cold stress (Ding et al., 2015). Therefore, the activity of OST1 is regulated by myristoylation and ubiquitination, which can be considered important mechanisms by which ICE1 accurately regulates plant cold tolerance. Moreover, in apple, the U-box-type E3 ubiquitin ligase MdPUB23 promotes the degradation of MdICE1 through the 26S proteasome pathway and negatively regulates cold tolerance (Wang et al., 2022). These studies have shown that phosphorylation, myristoylation and ubiquitination are involved in the transduction of cold signals and directly or indirectly regulate the ICE1-CBF-COR transcriptional cascade. Therefore, investigating the mechanism by which PTMs regulate cold signals will help elucidate the cold response mechanism of plants. However, the types of PTMs are abundant in plant cells (Zhang et al., 2022a), and it is not clear whether other PTMs regulate the ICE1-CBF-COR transcriptional cascade.

In addition to participating in growth and development, light signaling pathways are also involved in regulating *CBF* expression and responding to cold stress (Jiang et al., 2017, Jiang et al., 2020) (**Figure 3B**). Phytochrome interacting factors (PIFs) act as negative regulators that target *CBF*, with PIF3 binding to *CBF* promoters to downregulate their expression and negatively regulate cold tolerance. However, the E3 ubiquitin ligase ein3-binding f-box 1/2 (EBF1/2) can interact with PIF3 to promote its degradation through the 26S proteasome pathway (Jiang et al., 2017). In addition, phytochrome B (phyB) interacts with PIFs to precisely regulate *CBF* expression under long-day and short-day conditions (Lee and Thomashow, 2012). However, how phyB and PIFs perceive low-temperature signals is not clear. Interestingly, the feedback regulation of CBF, where CBF interacts with PIF3 to inhibit the codegradation of the PIF3 and phyB proteins, increases the stability of the phyB protein to promote the degradation of PIF1/4/5 via the 26S proteasome pathway and enhances the expression of the *COR* gene (Jiang et al., 2020). These studies have shown that the interactions among CBF, PIF and phyB integrate low temperature and light signals and play important roles in balancing plant photomorphogenesis and cold tolerance. Furthermore, elongated hypocotyl 5 (SIHY5), a bZIP-type light-temperature signal integration factor, not only directly regulates the expression of *CBFs* (Zhang et al., 2020a), but also indirectly increases the level of *CBF* transcripts by activating MYB and BBX in tomato (Zhang et al., 2020a; Song et al., 2023; Zhu et al., 2023). This information indicates that the integration of light and temperature signaling pathways is critical for *CBF* transcription.

The expression of cold tolerance genes is usually induced by cold stress, with the expression being inhibited at normal temperatures. Increasing evidence shows that the expression of *CBF* is also regulated by circadian clock factors (Kidokoro et al., 2023; Kim et al., 2024) (**Figure 3C**). In *Arabidopsis*, clock-related MYB TFs (RVE4/LCL1 and RVE8/LCL5) are rapidly transferred from the cytoplasm to the nucleus in response to cold stress, where they activate the expression of *DREB1s*. In contrast, the central oscillator components of the circadian clock, circadian clock associated 1 (CCA1) and late elongated hypocotyl (LHY), inhibit the expression of *DREB1s* under normal temperature (Kidokoro et al., 2021). In that process, cold stress enhances the phosphorylation of night light-inducible and clock-regulated (LNK) proteins and promotes the interaction between LNKs and RVEs to positively regulate *CBF* expression (Kidokoro et al., 2023). In addition, pseudoresponse regulators (PRRs), components of the circadian clock, negatively regulate the expression of *CBFs* via the circadian rhythm (Kidokoro et al., 2023). Interestingly, HOS15-mediated PRR7 degradation increases the expression of *CBF1* and *COR15A* under dark and cold stress (Kim et al., 2024). These evidences indicate that the circadian clock factors, as important switch for the expression of cold-responsive genes, accurately regulate the cold response of plants. However, it is not clear how circadian clock factors precisely regulate *CBF* and whether this regulation depends on other modification pathways. PTMs may represent a gap in understanding this pathway, as current evidence suggests that phosphorylation and ubiquitination mediated by LNK and HOS, respectively, are involved in this pathway.

CBF/DREB can bind to CRT/DRE (CCGAC) in the *COR* promoter to regulate the expression of *COR* genes and improve cold tolerance (Hwarari et al., 2022). During this process, DREB/CBFs need to interact with other proteins to enhance this regulation (Lee et al., 2021) (**Figure 3D**). For example, PbeNAC1 from *Pyrus betulifolia* interacts with PbeDREB1 to upregulate the transcription of *CORs* and increase cold tolerance in transgenic plants (Jin et al., 2017). In banana, the interaction between MaNAC1 and MaCBF1 enhances cold tolerance (Shan et al., 2014). In addition, thioredoxin h2 (Trx-H2) interacts with CBFs to modify the structure of CBF into monomers under cold stress, enhancing the regulation of *COR* expression by CBFs (Lee et al., 2021). Moreover, OST1 phosphorylates BTF3‐like protein (BTF3L, a β‐subunit of NAC) to facilitate the interaction of BTF3L with CBFs, increasing the stability of CBF under cold stress and thereby increasing the expression of *CORs* and cold tolerance in *Arabidopsis* (Ding et al., 2018). These studies have shown that interacting proteins are very important for the conformational changes and stability of CBF. However, the current understanding of CBF-interacting proteins is still insufficient. Generally, protein structure and stability are closely related to PTMs. Therefore, it is necessary to further study whether PTMs directly act on CBFs.

The ICE1-CBF-COR cascade is clearly a central pathway for the synergistic regulation of different signaling pathways to increase resistance to cold stress. In this complex network, various TFs contribute to the transcriptional level of the ICE1-CBF-COR cascade, and modifications at the protein level regulate its stability. The enhancement of the ICE1-CBF-COR cascade pathway may be one of the strategies for future genetic improvements in plant cold tolerance.

## ROS homeostasis regulates plant cold tolerance

**Figure 4:**
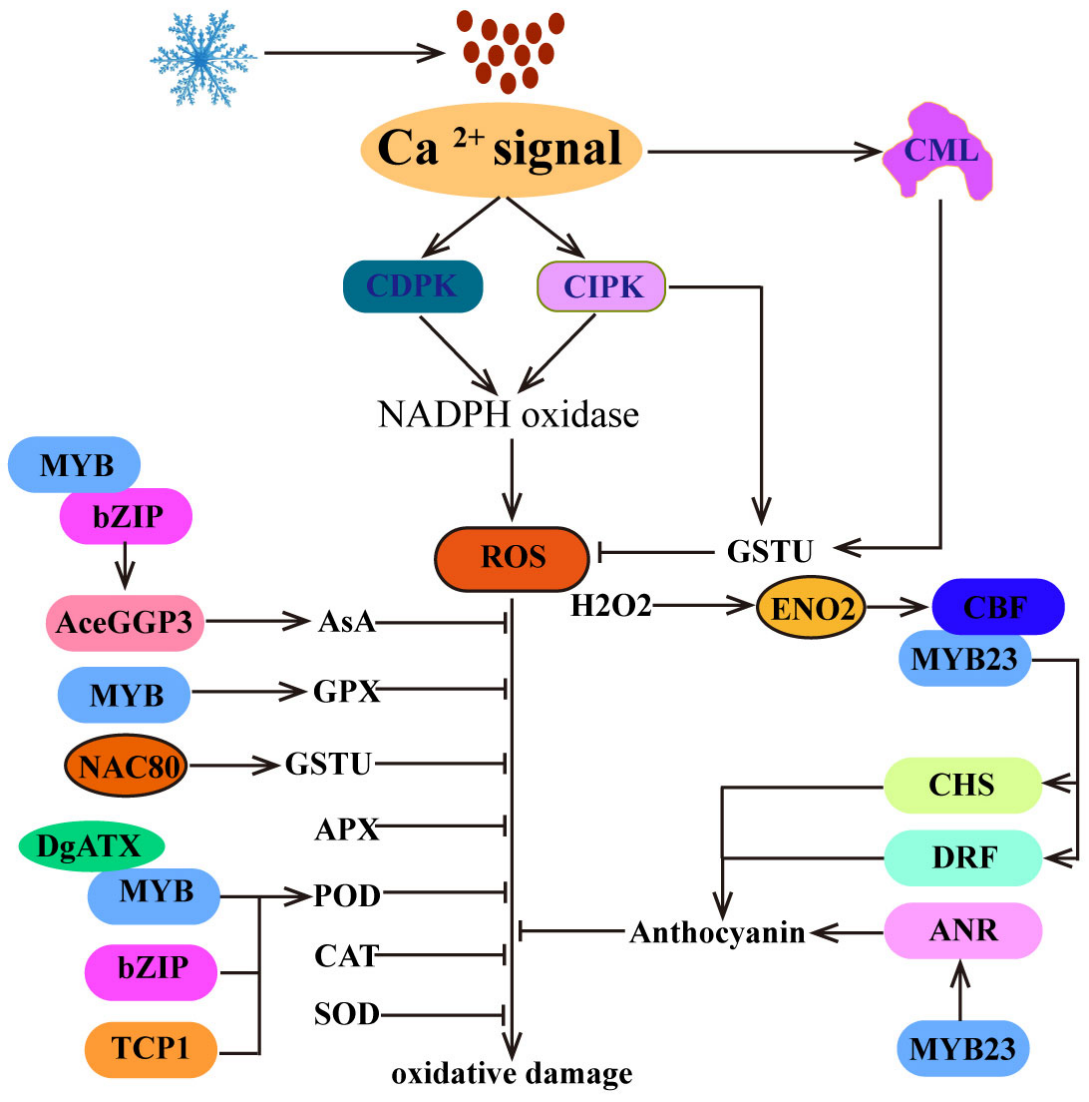
ROS homeostasis regulates plant cold tolerance. The Ca^2+^ signal is not only associated with the generation of early ROS signals but also promotes the clearance of excessive ROS. In addition, plants can scavenge excess ROS by increasing the activity of antioxidant enzymes, such as SOD, POD, CAT, GPX, APX, GSTU, and AsA. Moreover, H_2_O_2_, as a signaling molecule, positively regulates the expression of *CBF1*. Furthermore, CBF is involved in the biosynthetic pathway of anthocyanins and promotes ROS clearance.

Low temperatures can lead to the accumulation of reactive oxygen species (ROS), causing oxidative damage and cell death. To combat cold-induced damage, plants must effectively remove excess ROS. Typically, plants achieve this by increasing the activity of antioxidant enzymes (including SOD, POD, CAT, glutathione peroxidase (GPX), APX, and glutathione S-Transferase (GST)) (He et al., 2019; Ritonga and Chen, 2020; Yang et al., 2022a; Ye et al., 2024) and increasing the accumulation of antioxidants (such as L-ascorbic acid (AsA), Glutathione (GSH) and anthocyanin) (Zhou et al., 2020; Ma et al., 2021; Liu et al., 2023a). The physiological activity and level of antioxidant enzymes are usually induced by cold stress, which is important for ROS scavenging. In this process, TFs such as bZIP, MYB, TCP, and NAC bind to the promoters of genes that encode antioxidant enzymes, thereby promoting their expression to remove excessive ROS (**Figure 4**). For example, cold-induced AcePosF21 (a bZIP TF) interacts with AceMYB102, directly binds to the promoter of GDP-L-galactose phosphorylase 3 (*AceGGP3*), a key regulatory gene in AsA biosynthesis, and promotes AsA production, thereby eliminating excessive ROS produced by chilling injury and improving cold tolerance in kiwifruit (Liu et al., 2023a). In *chrysanthemum*, DgMYBs directly target the promoters of *GPX* and *POD* to increase the activity of GPX and POD and reduce the accumulation of ROS (Yang et al., 2022a; Luo et al., 2024b). Similarly, DgbZIP3 and DgTCP1 are capable of binding to the promoter of *DgPOD* to promote the expression of *DgPOD* and reduce the accumulation of ROS, thus improving cold tolerance in *chrysanthemum* (Bai et al., 2022; Li et al., 2022b). In addition, the translocation of de-S-acylated MtNAC80 to the nucleus is mediated by acyl protein thioesterases (MtAPT1), and MtNAC80 subsequently binds to the promoter of *MtGSTU1* and promotes its expression, thereby reducing the accumulation of H_2_O_2_ and improving the cold resistance of *Medicago truncatula* (Ye et al., 2024). These studies have shown that TFs are critical for the regulation of antioxidase activity. Therefore, further exploration of cold-inducible TFs that target antioxidant enzyme activities will help to elucidate the antioxidant protection mechanism of plants under cold stress.

Moreover, cold stress can also induce the biosynthesis of anthocyanins to eliminate the accumulation of ROS, thereby increasing cold tolerance in plants (**Figure 4**). In this process, MYBs bind to the promoters of anthocyanin biosynthesis-related genes (chalcone synthase (*CHS*), dihydroflavonol 4-reductase (*DRF*) and anthocyanidin reductase (*ANR*)) to promote their expression and anthocyanin biosynthesis. For example, MdMYB23 binds to the promoter of *MdANR* and activates its expression to promote proanthocyanidin accumulation and ROS scavenging (An et al., 2018). In addition, SmCBFs interact with SmMYB113 to regulate the expression of *SmCHS* and *SmDRF*, promoting anthocyanin biosynthesis and ROS scavenging and thereby increasing cold tolerance in transgenic plants (Zhou et al., 2020). Interestingly, in strawberry, FveDREB1B promotes anthocyanin accumulation by directly activating *FveCHS* (Luo et al., 2024a). These studies have shown that CBF directly or indirectly regulates the cold-induced anthocyanin biosynthesis pathway and promotes ROS scavenging.

Previous studies have shown that cold stress-induced ROS production is derived from nicotinamide adenine dinucleotide phosphate-oxidase (NADPH oxidase) in plant cells (Kawarazaki et al., 2013). The activity of NADPH oxidase is regulated by CDPK and CIPK (Kobayashi et al., 2007; Kimura et al., 2013) (**Figure 4**). These findings indicate that Ca^2+^ is closely related to the production of early ROS signals. Interestingly, in *Medicago sativa*, MsCML10 is activated by cold-induced Ca^2+^ signaling and interacts with MsGSTU8 to maintain ROS homeostasis and enhance cold tolerance (Yu et al., 2022). In *Camellia sinensis*, CsCIPK11 phosphorylates CsGSTU23, enhances its stability and positively regulates cold tolerance (Di et al., 2024). These findings suggest that Ca^2+^ signaling plays a crucial role in the regulation of ROS homeostasis.

While the accumulation of ROS in plants can potentially result in oxidative damage, various studies have shown that ROS also play a crucial role in transmitting signals related to abiotic stress (You and Chan, 2015). In *Arabidopsis*, increasing H_2_O_2_ levels can improve cold tolerance, whereas decreasing H_2_O_2_ contents can diminish cold tolerance (**Figure 4**). This phenomenon is attributed to the fact that cold-induced H_2_O_2_ increases the nuclear import of cytosolic enolase 2 (ENO2) by sulfenylating cysteine 408, allowing it to directly bind to the promoter of the *CBF1* gene and activate its expression, enhancing cold stress tolerance (Liu et al., 2022). Although there is currently no more evidence to explain the relationship between *CBF* and ROS, these findings emphasize the importance of H_2_O_2_ as a signaling molecule in regulating the expression of *CBF*, indicating an indirect regulatory relationship between *CBF* and ROS homeostasis.

## Response of hormone signaling pathways to cold stress

**Figure 5:**
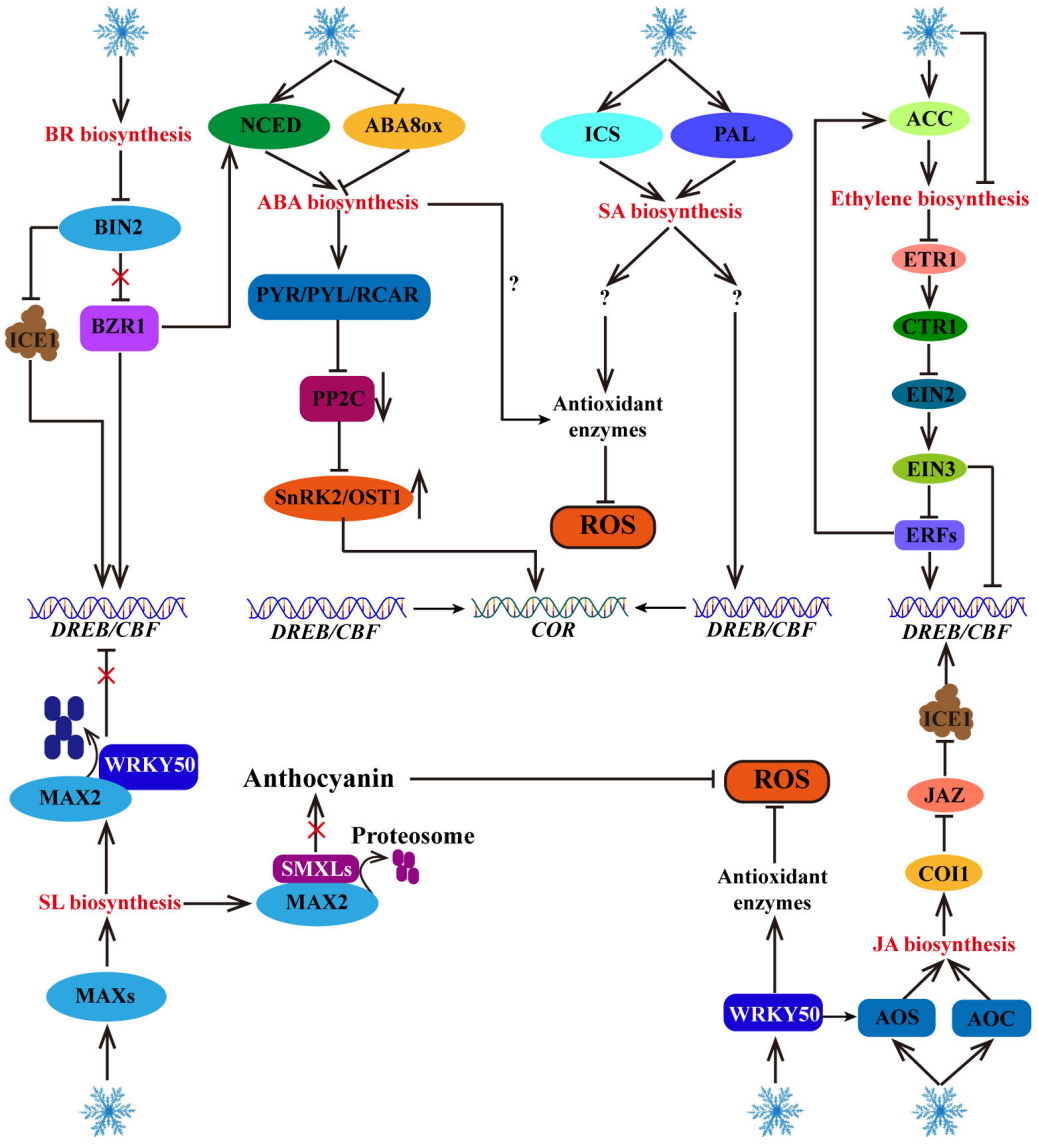
Response of hormone signaling pathways to cold stress. Cold stress increases the biosynthesis of plant hormones, including ABA, BR, JA, SA, ethylene and SL. These plant hormones further activate their signaling pathways to directly or indirectly regulate the CBF-COR cascade. In addition, some plant hormones (such as ABA, SA, SL and JA) contribute to ROS scavenging.

Abscisic acid (ABA) is a plant hormone that plays an important role in plant growth, development and stress response. It is derived from zeaxanthin, a process catalyzed by the ABA1 (zeaxanthin epoxidase), ABA4 (neoxanthin synthase), NCED (9-cis-epoxycarotenoid dioxygenase), ABA2 (xanthoxin dehydrogenase) and ABA3 (molybdenum cofactor sulfurase) enzymes in five steps (Hauser et al., 2017). Many studies have shown that appropriate regulation of exogenous or endogenous ABA levels plays an important role in plant cold stress responses (Miura and Nozawa, 2014; Mega et al., 2015; Zhang et al., 2019b; Lim and Lee, 2020; Cai et al., 2021; Guo et al., 2021; Shen et al., 2022). Exogenous ABA treatment can reduce the damage caused by low temperature to cell membranes, increase the endogenous ABA content and improve the cold tolerance in sugarcane (Huang et al., 2015). Similarly, exogenous ABA treatment can increase the activities of POD, CAT, and APX, reduce membrane lipid peroxidation damage and induce endogenous ABA accumulation, and increase cold tolerance in melon (Li et al., 2021). These studies indicate that ABA positively regulates cold tolerance in plants. Cold stress induces the expression of the ABA biosynthesis-related gene *NCED* and downregulates the ABA degradation gene *CYP707A* (encoding ABA 8’-hydroxylase), thereby increasing the content of endogenous ABA and improving cold tolerance in *Fragaria* species (Shen et al., 2022). The overexpression of *NCED3* in melon promotes ABA accumulation and triggers the expression of ABRE-binding factor (*ABFs*), which enhances cold tolerance (Li et al., 2022a). The ABA-responsive element binding protein/ABRE-binding factor (AREB/ABF) is a key TF downstream of ABA signaling. Exogenous ABA treatment and the accumulation of endogenous ABA can increase *ScAREB4* transcription, leading to increased cold tolerance in potato plants (Liu et al., 2023b). ABA is oxidized by the ABA8ox enzyme (mainly encoded by the *CYP707A1*, *CYP707A2*, *CYP707A3* and *CYP707A4* genes) into phaseic acid (PA), which is further reduced to inactive dihydrophaseic acid (DPA) (Cutler and Krochko, 2000; Dong et al., 2015). The overexpression of the *OsABA8ox1* can reduce the endogenous ABA content and increase the sensitivity of rice to low-temperature stress (Mega et al., 2015). These studies revealed that increasing ABA biosynthesis and preventing ABA catabolism can increase plant cold tolerance. Furthermore, ABA can bind to the Pyrabactin resistance (PYR)/pyrabactin resistance-like (PYL)/regulatory component of ABA receptors (RCAR) receptors, inhibit the activity of protein phosphatase 2C (PP2C), maintain the phosphorylation of SnRK2/OST1 protein kinases, and activate the expression of downstream cold response genes (*CORs*) (Ma et al., 2009; Boneh et al., 2012; Lim and Lee, 2020). These studies revealed that the ABA biosynthesis pathway gene *NCED* is upregulated, whereas the ABA degradation gene *CYP707A* is downregulated in response to cold stress, resulting in endogenous ABA accumulation. Furthermore, ABA binds to PYR/PYL/RCAR receptors, inhibiting PP2C protein phosphatase activity and activating SnRK2/OST1 protein kinases (sucrose-non-fermenting-1-related protein kinase 2) to regulate the expression of downstream cold response genes, ultimately increasing plant cold tolerance (**Figure 5**). These studies fully demonstrate that ABA positively regulates plant cold tolerance independent of CBF.

Brassinosteroid (BR) is an important plant hormone that play important roles in the cold stress response. Cold-induced DWARF (*DWF*, a BR synthesis gene) and brassinazole resistant 1 (*BZR1*, a positive regulator of the BR signaling pathway), can increase *NCED1* gene expression to increase the level of ABA, thereby enhancing cold tolerance in tomato plants (An et al., 2023). In *Arabidopsis*, *BZR1* positively regulates freezing tolerance through CBF-dependent and CBF-independent pathways: low temperature promotes the dephosphorylation of BZR1 to regulate *CBF1/CBF2* expression. In addition, dephosphorylated BZR1 regulates the expression of cold response genes (such as *WKRY6*, *PYL6*, suppressor of overexpression of constans1 (*SOC1*), jasmonic acid carboxyl methyltransferase (*JMT*) and Senescence Associated Gene 21 (*SAG21*)) through a CBF-independent pathway (Li et al., 2017). Brassinosteroid-insensitive 2 (BIN2) is a negative regulator of the BR signaling pathway that can inhibit BZR1 and CBF, thereby negatively regulating cold tolerance. Cold stress and BR can inhibit the transcription of BIN2 protein kinase, thereby increasing the transcription level of *BZR1* and promoting the expression of the *NCED1* gene to increase ABA accumulation in tomato plants (An et al., 2023). Additionally, BIN2 can phosphorylate ICE1, facilitating the interaction of ICE1 with the E3 ubiquitin ligase HOS1, thereby promoting ICE1 degradation and negatively regulating *CBF* gene expression in *Arabidopsis* (Ye et al., 2019). These studies have shown that BR positively regulates plant cold tolerance through the ABA and CBF pathways (**Figure 5**).

As an important plant hormone, salicylic acid (SA) not only regulates plant defense immunity but also promotes plant cold tolerance. The synthesis of endogenous SA is derived from the isochorismate synthase (ICS) and phenylalanine ammonia-lyase (PAL) pathways. Cold stress can induce these two pathways and promote SA accumulation (Kim et al., 2013; Dong et al., 2014). Many studies have shown that under low-temperature stress, exogenous SA treatment and endogenous SA accumulation can improve plant antioxidant enzyme activity and ROS scavenging ability, promote the expression of cold response genes, such as *WRKY*, *CBFs*, and *CORs*, and increase plant cold tolerance (Ding et al., 2016; Aazami and Mahna, 2017; Pan et al., 2020; Wang et al., 2020; Li and Wang, 2021). Although SA positively regulates plant cold tolerance through the CBF-dependent pathway and ROS homeostasis regulation, how does SA activate CBF? How can the activity of antioxidant enzymes be promoted? These regulatory mechanisms are not yet clear (**Figure 5**).

Jasmonic acid (JA) is an important plant hormone involved in the plant response to cold stress. Exogenous treatment with exogenous JA has been shown to increase plant cold tolerance, and the biosynthesis of endogenous JA is activated under cold stress (Hu et al., 2017). In tomato, cold stress-induced SlWRKY50 can bind to the promoter of the allene oxide synthase (*SlAOS*) gene, a vital enzyme in the JA synthesis pathway, to positively regulate its transcription and facilitate JA biosynthesis (Wang et al., 2023a). In addition, cold stress induces the expression of allene oxide cyclase (*MfAOC2*), another key enzyme in JA biosynthesis, to promote the accumulation of JA, which increases the expression levels of *CBF* genes and enhances cold tolerance in *Medicago truncatula* (Yang et al., 2023). Moreover, under normal temperatures, jasmonate zim-domain (JAZ), an inhibitor of the JA signaling pathway, can interact with ICE1/2 to inhibit the transcriptional activity of *ICE1*. However, under cold stress conditions, the JA receptor coronatine insensitive 1 (COI1) can degrade JAZs, thereby activating the transcriptional activity of *ICE1*, promoting *CBF* gene expression, and improving cold tolerance in *Arabidopsis* (Hu et al., 2013). These findings demonstrate that cold stress induces the expression of genes involved in JA biosynthesis, leading to JA accumulation and the positive regulation of plant cold tolerance through the ICE1-CBF-COR pathway (**Figure 5**).

Strigolactones (SLs) are carotenoid phytohormones that not only participate in plant morphogenesis but also play important roles in the cold stress response (Cooper et al., 2018). Cold stress triggers the expression of more axillary growth (*MAX*) genes to promote the accumulation of SLs (Chi et al., 2021). Furthermore, SLs positively regulate frost resistance through the CBF-dependent pathway (SLs promote the interaction between MAX2 and WRKY41, mediate the degradation of WRKY41 through the 26S proteasome pathway, and relieve the inhibitory effect of WRKY41 on the expression of *CBFs*) and the CBF-independent pathway (SLs facilitate the interaction between MAX2 and suppressor of max2‐like (SMXLs), increase the degradation of SMXLs, and promote the accumulation of anthocyanin) in *Arabidopsis* (Wang et al., 2023b) (**Figure 5**).

Ethylene plays dual roles in the cold stress response of various plants (**Figure 5**). In tomato, cold-induced *SlNAM3* activates the transcription of *ACC* (1-aminocyclo-propane-1-carboxylic acid) synthase genes (1-Aminocyclopropane-1-carboxylic acid synthase (*SlACS1*) and 1-aminocyclopropane-1-carboxylic acid oxidase (*SlACO*)) by directly binding to their promoters to promote ethylene production and increase cold tolerance, indicating that ethylene positively regulates cold tolerance (Dong et al., 2022). In contrast, in Arabidopsis, endogenous ethylene overproduction and exogenous ACC treatment can reduce cold tolerance under plate culture conditions, but cold tolerance is increased by the application of an ethylene biosynthesis inhibitor, indicating that high concentrations of ethylene are not conducive to cold tolerance in *Arabidopsis* (Shi et al., 2012). In addition, cold stress inhibits ethylene biosynthesis and negatively regulates the ethylene signaling pathway (ACS-ethylene-ethylene receptor (ETR)-constitutive triple response 1 (CTR1)-ethylene insensitive 2 (EIN2)-EIN3-CBF) to increase the transcription level of *CBF*, indicating that the ethylene signaling pathway negatively regulates *CBF* in *Arabidopsis* (Shi et al., 2012). However, it has been found that maintaining adequate levels of endogenous ethylene also contributes to resistance to cold stress in *Arabidopsis*. For example, under potting soil conditions, cold-induced rare cold inducible 1a (RCI1A) interacts with ACS to decrease ACS stability, leading to a decrease in ethylene biosynthesis and cold tolerance in *Arabidopsis* (Catala et al., 2014). Since ethylene is a volatile gas, high-humidity plate culture conditions may inhibit the release of ethylene. Unlike in *Arabidopsis*, the cold tolerance of apple increases after treatment with exogenous ACC but decreases after the application of an ethylene biosynthesis inhibitor (Wang et al., 2021a). Under cold stress, MdERF1B, an ethylene-responsive factor, binds to the promoter of *MdACO1* to promote its expression and increase ethylene production, and its cold tolerance further improves via ERF-mediated activation of *CBF* expression (Wang et al., 2021a). Moreover, cold-induced PtrERF9 enhances ethylene biosynthesis by activating PtrACS1, thereby positively regulating cold tolerance in *Poncirus trifoliata* (Zhang et al., 2022b). These studies highlight the role of ERF positive feedback in regulating the cold-induced ethylene signaling pathway, and also indicate that ERF-mediated ethylene signaling regulates *CBF* transcription.

In summary, plant hormones, including ABA, BR, JA, SA, ethylene, and SL, play crucial roles in the response of plants to cold stress. These hormones are involved in CBF-dependent pathways and also contribute to ROS scavenging. The response of the ethylene signaling pathway to cold stress appears to vary among different plant species. However, how cold stress signals activate plant hormone signaling pathways remains unclear.

## Conclusion and future perspectives

The response of plants to cold stress is a typical quantitative genetic trait involving the regulation of multiple genes. Cold stress signals are detected by plants through cold sensors, and the transduction and amplification of these signals are mediated by Ca^2+^ signaling and protein kinase pathways. The ICE1-CBF-COR transcriptional cascade is a key pathway activated to combat low temperature stress. This process involves various factors such as TFs, PTMs, light signals, circadian clock factors, and interacting proteins. Additionally, ROS homeostasis and plant hormone signaling pathways play important roles in the response to cold stress in both CBF-dependent and CBF-independent manners, highlighting the interconnected nature of the ICE1-CBF-COR cascade, ROS homeostasis, and plant hormone signaling.

It is evident from the existing studies that the plant response to cold stress involves a complex regulatory network with interconnected signaling pathways. To gain a better understanding of this network, it is crucial to elucidate the molecular mechanisms underlying the interactions involved in the cold tolerance pathways. Moreover, PTMs, such as phosphorylation, ubiquitination and myristoylation, play a role in regulating TFs and cold-tolerant proteins under cold stress. Further integration of multiomics data, including genome, transcriptome, proteome, and PTMs data, will be essential for a comprehensive investigation of the cold stress regulatory network in plants.

Many studies have shown that manipulating the expression of a few genes can affect the cold tolerance of plants, particularly during the seedling stage under ideal conditions. However, further research is needed to investigate whether these genes can increase cold tolerance throughout the entire growth period in natural environments. Research has indicated that the overexpression of genes related to cold tolerance can lead to a reduction in the biological yield of transgenic plants due to increased energy consumption. For example, the overexpression of *DREB/CBF* genes in *Arabidopsis* and rice resulted in dwarf phenotypes (Liu et al., 1998; Ito et al., 2006). This phenomenon is attributed to the constitutive expression of cold tolerance genes, which necessitates energy expenditure. Therefore, the identification of cold-inducible promoters that regulate the expression of cold-tolerant genes, allowing plants to activate these genes selectively in response to cold stress, represents a crucial approach for the future breeding of cold-tolerant crop varieties.

## Glossary

ABA: abscisic acid
ACO: 1-aminocyclopropane-1-carboxylic acid oxidase
ACS: 1-Aminocyclopropane-1-carboxylic acid synthase
ANNEXIN1: Ca2+-permeable transporter
ANR: anthocyanidin reductase
AOC: allene oxide cyclase
AOS: allene oxide synthase
AP2/ERF: APETALA2/ethylene- responsive element binding factor
APT: acyl protein thioesterases
AREB/ABF: ABA-responsive element binding protein/ABRE-binding factor
AsA: L-ascorbic acid
BBX: B-box
bHLH: basic helix-loop-helix
BIN2: brassinosteroid insensitive 2
BR: brassinosteroid
bZIP: basic leucine zipper
BZR1: brassinazole resistant 1
CaM/CML: calmodulin/calmodulin-like protein
CAMTA: calmodulin-binding transcription activator
CBF: C-repeat binding factor
CBL: calcineurin B-like protein
CCA1: circadian clock associated 1
CDPK/CPK: calcium-dependent protein kinase
CHS: chalcone synthase
CIPKs: CBL protein kinases
CNGCs: cyclic nucleotide-gated channels
COG3: Geng/japonica rice 3
COI1: coronatine insensitive 1
COLD1: chilling-tolerance divergence 1
*COR*: cold-reulated gene
CRLK1: Ca2+/CaM-regulated receptor-like kinase 1
CRPK1: cold-responsive protein kinase 1
CRT3: calreticulin 3
CTR: constitutive triple response
CYP707A: ABA 8’-hydroxylase
DRF: dihydroflavonol 4-reductase
EBF1/2: ein3-binding f-box 1/2
EGR2: clade-E growth-regulating 2
EIN: ethylene insensitive
ENO2: cytosolic enolase 2
ETH: ethylene
ETR: ethylene receptor
GGP3: GDP-L-galactose phosphorylase 3
GPX: glutathione peroxidase
GSH: Glutathione
GST: glutathione S-Transferase
HOS1: high expression of osmotically responsive gene 1
ICE1: inducer of CBF expression 1
ICS: isochorismate synthase
JA: jasmonic acid
JAZ: jasmonate zim-domain
JMT: jasmonic acid carboxyl methyltransferase
LHY: late elongated hypocotyl
LNK: night light-inducible and clock-regulated
MAPK: mitogen-activated protein kinase
MAX: more axillary growth
MCA1: mid1 complementing activity
MYB: myeloblastosis
NAC: (no apical meristem (NAM), *Arabidopsis* transcription activation factor (ATAF) and cup-shaped cotyledon (CUC))
NADPH: nicotinamide adenine dinucleotide phosphate
NCED1: 9-cis epoxycarotenoid dioxygenase
NLP7: nin-like protein 7
NMT1: N-myristoyltransferase 1
SERL2: somatic embryogenesis receptor-like kinase 2
OST1: open stomata 1
PAL: phenylalanine ammonia-lyase
phyB: phytochrome B
PIF: phytochrome interacting factors
PP2C: protein phosphatase 2C
PRRs: pseudoresponse regulators
PSY1R: sulfated tyrosine1 receptor
PTMs: post-translational modifications
RCI1A: rare cold inducible 1a
RG4: RNA G-quadruplex
RGA1: G-protein α
ROS: reactive oxygen species
RVE: REVEILLE
SA: salicylic acid
HY5: elongated hypocotyl 5
SL: strigolactones
SMXLs: suppressor of max2‐like
SnRK2: sucrose-non-fermenting-1-related protein kinase 2
SOC1: constans1
TCP: teosinte branched 1/cycloidea/proliferating cell factor
TFs: transcription factors
Trx-H2: thioredoxin h2
